# Treating Postlaparoscopic Surgery Shoulder Pain with Acupuncture

**DOI:** 10.1155/2014/120486

**Published:** 2014-04-23

**Authors:** Gur Kreindler, Samuel Attias, Anna Kreindler, Haim Hen, Bassel Haj, Ibrahim Matter, Eran Ben-Arye, Elad Schiff

**Affiliations:** ^1^Complementary and Integrative Surgery Service, Bnai-Zion Medical Center, 47 Golomb Street, 31048 Haifa, Israel; ^2^School of Public Health, University of Haifa, Mount Carmel, 31905 Haifa, Israel; ^3^Faculty of Management, Tel-Aviv University, P.O. Box 39040 , 69978 Tel-Aviv, Israel; ^4^Department of General Surgery, Bnai-Zion Medical Center, 47 Golomb Street, 31048 Haifa, Israel; ^5^Complementary and Traditional Medicine Unit, Department of Family Medicine, Faculty of Medicine, Technion International School, Mauerberger building, 2nd floor, Technion City, 3200003 Haifa, Israel; ^6^Department of Internal Medicine, Bnai-Zion Medical Center, 47 Golomb Street, 31048 Haifa, Israel; ^7^The Department of Complementary/Integrative Medicine, Law and Ethics, The International Center for Health, Law and Ethics, Haifa University, 199 Aba Khoushy Avenue, Mount Carmel, Haifa, Israel

## Abstract

*Objective.* The purpose of this study was to examine the effect of acupuncture on postlaparoscopic shoulder pain (PLSP) which is a common side effect in patients undergoing abdominal laparoscopic surgery. *Methods.* Patients with moderate to severe PLSP in spite of analgesic treatment, which were referred by the medical staff to the Complementary-Integrative Surgery Service (CISS) at our institution, were provided with acupuncture treatment. The severity of PLSP and of general pain was assessed using a Visual Analogue Scale (VAS) from 0 to 10. Pain assessment was conducted prior to and two hours following acupuncture treatment. Acupuncture treatment was individualized based on traditional Chinese medicine diagnosis. *Results.* A total of 25 patients were evaluated during a 14-month period, from March 2011 to May 2012. A significant reduction in PLSP (mean reduction of 6.4 ± 2.3 P < 0.0001) and general pain (mean reduction 6.4 ± 2.1 P < 0.0001) were observed, and no significant side effects were reported. *Conclusion.* Individualized acupuncture treatments according to traditional Chinese medicine principles may improve postlaparoscopic shoulder pain and general pain when used in conjunction with conventional therapy. The primary findings of this study warrant verification in controlled studies.

## 1. Introduction

Laparoscopic surgery, also called minimally invasive surgery, is a modern surgical technique in which operations in the abdomen are performed through small incisions so that a miniature video camera can be inserted and allow the surgeon to examine the surgical area [1]. In order to enhance the surgeon's ability to clearly view the operating area, the abdomen is inflated using carbon dioxide (CO_2_) [1, 2]. The inflation of CO_2_ is believed to cause shoulder pain, which is often associated with laparoscopic procedures, due to irritation of the diaphragm. The incidence of shoulder pain varies from 35% to 80%, ranges from mild to severe, and usually lasts up to 72 hours after surgery [1, 2]. 38% of patients report constant pain in the 48–72 hrs following surgery, while 28% experience it intermittently. Most patients will require analgesics or nonpharmacologic (heat packs, etc.) treatment for pain relief [3].

Current treatment of postlaparoscopic shoulder pain (PLSP) is not standardized and may include preventive measures such as removing CO_2_ by means of Trendelenburg position (the body is laid flat on the back, with the feet higher than the head by 15–30 degrees) and a pulmonary recruitment maneuver [2, 4], intraperitoneal gas drain [4], use of warmed and humidified insufflations of carbon dioxide [5, 6], and pain control after surgery with analgesic medications [7–9]. However, these treatments are often unsatisfactory or carry side effects [10].

Integration of complementary medicine (CM) is being used increasingly in medical settings [11, 12], especially for various pain syndromes [13]. In 2010 a unique service integrating CM in the Surgery Department at the Bnai-Zion Medical Center in Israel was established (Complementary and Integrative Surgery Service (CISS)). Practitioners at the CISS provide various CM treatments to palliate common perioperative symptoms. Treatment outcomes are documented and analyzed via a rigorous registry protocol [14, 15]. Acupuncture was reported in several studies as an effective treatment for reducing postoperative pain [16], and therefore we considered it as potentially appropriate for PLSP. During the first months of the CISS's activity, practitioners developed a traditional Chinese medicine (TCM) based approach for treating patients with PLSP. The treatment approach was developed through a process which included conducting a literature review of conventional medicine and classical TCM texts relevant to PLSP; observation of multiple laparoscopic surgeries; acupuncturists' group discussions on the topic; and extensive clinical exploration of the various approaches that were suggested [17]. When a consensus among the practitioners was reached regarding the treatment approach, we began gathering data on the outcomes systematically. In this paper we report the outcomes of patients experiencing PLSP that were referred to our service by the medical staff (physicians and nurses) between March 2011 and May 2012. This is the first paper in the English literature reporting on acupuncture treatment for PLSP.

## 2. Methods

The study was conducted at the Bnai-Zion Medical Center in the General Surgery Department from March 2011 to May 2012. The study was approved by the institution's IRB and was registered with ClinicalTrials.gov (NCT01733771).

Inclusion criteria consisted of patients who experienced clinically significant PLSP (reporting pain of more than 3 on a Visual Analogue Scale of 0–10) which appeared within 24 hrs following laparoscopic surgery (mainly cholecystectomy, hernias in the anterior abdominal wall, and bariatric surgery) and did not respond satisfactorily to analgesic use. Analgesics were provided according to the departments' pain management protocol. If within two hours of analgesic provision pain persisted with a VAS score of four and above, patients were referred to acupuncture. Exclusion criteria included age under 16 and first trimester of pregnancy.

Patients were categorized according to classical TCM clinical patterns/syndromes [18–20]. The TCM diagnosis was based on a thorough physical examination including traditional tongue and pulse diagnosis as well as facial and voice diagnosis [21]. TCM diagnosis of patients with PLSP was validated by two TCM practitioners (each with a minimum of seven years in practice). Whenever there were disagreements regarding the TCM pattern categorization, a third TCM practitioner was involved until resolution was achieved. Based on the individualized TCM diagnosis, a customized acupuncture treatment plan was formulated.

Acupuncture treatments were documented according to STRICTA guidelines [22]. Disposable soft spring handle needles (Dongbang Acupuncture Inc.) 20 × 30 mm were utilized. After insertion of the needles and achieving a “De Qi” sensation (warmth, numbness around the needle), needles were stimulated according to the TCM syndrome [23]. The needles were left in place according to the particular needle technique employed:* dispersing* for 30 minutes,* balancing* for 20 minutes, and* strengthening *for 15–18 minutes. The number of points chosen in each treatment ranged between four and eight. Some points were placed bilaterally and others on one side. For a detailed description of the acupuncture points that were used according to each syndrome, see Table 5.

Shoulder pain was evaluated using Visual Analogue Scale (VAS) with a scale of “no symptom whatsoever” to “unbearable symptom severity” (100 mm length line). There were no numbers on the scale. The questionnaires were presented on a touch screen, utilizing iPAD application technology. Patients reported symptom severity on the VAS scale prior to treatment and within 2 hours following it. Each patient was provided with the first acupuncture treatment within 24 hours of surgery. The degree of shoulder pain relief was considered clinically significant if a reduction of more than 15 mm was reported on VAS.

## 3. Statistical Analysis

Data was entered and managed using the Microsoft Excel 2003 Program and later analyzed using the SPSS Program, version 18 (IBM SPSS, Chicago, IL, USA). The normality of the data was tested by Kolmogorov-Smirnov *Z*. As the pain variables were normally distributed, paired *t*-test was conducted for differences between before and after acupuncture treatments. Pearson's correlation was conducted for determining the strength and direction of the association between general pain and shoulder pain and also to measure the linear association between those two variables. *P* ≤ 0.05 was considered significant.

## 4. Results

### 4.1. Patient Referral and Characteristics

Twenty-six patients were referred to acupuncture treatment for PLSP by the surgery departments' physicians and nurses. One patient was not willing to participate in the study due to fear from needles. Twenty-five patients provided consent for acupuncture treatment and received individualized acupuncture treatment for PLSP. Two patients received an additional treatment for PLSP on the consecutive day. Patient characteristics are presented in Table 1.

### 4.2. Pain Measurement

Changes in PLSP are presented in Tables 2 and 4. The mean pain for PLSP before treatment was 8.3 ± 1.8 on the VAS. PLSP severity before intervention ranged from 3.6 to 9.9. Two hours following treatment mean pain for PLSP decreased to 1.8 ± 1.8 (*P* < 0.0001) with a mean reduction of 6.4 ± 2.3. PLSP severity 2 hours following intervention ranged from 0.2 to 6.6.

We also evaluated postoperative pain (general pain) around the operated area, as presented in Tables 3 and 4; patients were requested to report their general pain (not associated with PLSP) on VAS scales at the same time they were evaluated for PLSP. The acupuncture treatments were provided with the goal of relieving general pain as well as PLSP. In 24 patients the mean reduction of pain was 6.4 ± 2.5 (*P* < 0.0001).

In addition, we found a linear correlation between mean difference in general pain and mean difference in shoulder pain (Pearson *r* = 0.512, *P* = 0.013), meaning that patients who had a greater reduction in PLSP had a similar reduction in general pain and vice versa.

### 4.3. Adverse Events

We asked patients to report adverse effects associated with the treatment such as hematomas, needle pain, nausea, and weakness. We assessed fainting and forgotten needles. No adverse events were reported during this trial by patients and practitioners.

### 4.4. PLSP Categorization into TCM Syndromes

We identified five main TCM syndromes in patients with PLSP and acupuncture points relevant to the treatment of each syndrome (Tables 4 and 5).

#### 4.4.1. Phlegm

People undergoing a surgical process are subjected to a world full of emotions. Threat of death, familial relations, and personal emotional and spiritual resources are at an extreme.

Excess of any of the five emotions in TCM, that is, anger, fear/fright, happiness, worry/doubt, and sadness, leads to an abundance of Qi. Excess Qi then transforms into “fire,” which condenses the body fluids and forms phlegm [24].

Anxiety and fright are the most common emotions during presurgical preparations. Gong Ju Zhong, a famous TCM scholar, said that “Phlegm is produced by fright. The Shen (spirit) leaves its residence, and when the residence is empty, the fluids will form phlegm” [24]. In turn, phlegm blocks Qi, consequently causing pain as a result of Qi blockage. The treatment protocol we chose for treating phlegm had two objectives: (i) to dispel and disperse the phlegm that was produced and (ii) to stop the present formation and prevent the future formation of phlegm.

In 15 of the cases phlegm was part of the syndrome. We found that, by choosing points that disperse phlegm, patients first described a sense of emotional relief, followed by reduction of pain.

#### 4.4.2. Qi and Blood Stagnation

The formation of pain in the shoulder after laparoscopic surgery was referred to as Qi and/or blood stagnation. This was diagnosed based on the combination of two criteria—the presence and intensity of the purple color on the patient's tongue and the characteristics of sharp and severe pain [18–21]. This form of stagnation may be generated by the emotional stress of undergoing a surgical procedure, as well as the mechanics of the surgical procedure itself.

#### 4.4.3. Heat

In 14 patients heat was observed. Two parameters were used to diagnose heat: tongue and pulse diagnosis. Tongue diagnosis of heat was based on findings such as the appearance of red dots on the tongue, with their position indicating in which burner (upper, middle, or lower burner) the heat is accumulating [18–20]. Another important finding was the appearance of yellow coating on the tongue [18].

The second parameter used to determine heat was the pulse. An indication of heat would be a rapid pulse [19, 25]. A secondary tool used to assess heat was facial diagnosis. Rosy cheeks or any form of redness on the face would indicate heat [19–21]. We found that heat would appear in patients who often reported emotional frustration, had a harder time dealing with change, or were lonely for long periods of time. Following acupuncture, patients reported being calmer and the medical staff noticed that they were more compliant and easier to deal with.

#### 4.4.4. Dampness

Dampness was diagnosed in 4 patients according to three major characteristics: having a slippery/wavy pulse [19, 25]; being obese or having a subjective feeling of “heaviness” in the body; having a swollen tongue with white or yellow coating, where the thickness of the coating would indicate the severity of the dampness [18–21]. These patients were less willing to “get up and move around”; this may have hampered their rehabilitation. The aim of the treatment was to mobilize them and resolve dampness, thereby reducing pain and facilitating movement.

#### 4.4.5. Deficiency Syndromes

The deficiency is manifested in the tongue and pulse, which generally look less vital [18, 19, 25]. Patients often exhibit a white or yellowish complexion. Their voice may be very weak and it is hard for them to conduct a lengthy conversation. Some patients even found it hard to fill out the questionnaires. The detailed diagnostics of deficiency syndromes are well known in the classical TCM literature [18–21, 23–25].

In our case series, we found that patients often suffered from Qi deficiency. Nine patients suffered from spleen Qi deficiency. This could be due to the lifestyle they conducted before the surgery. It may also have been caused by the prolonged fasting before and after surgery.

One patient suffered from lung Qi deficiency; this was attributed to heavy smoking and lack of exercise or diet.

Five patients suffered from Yin deficiency involving the kidney and the stomach. These patients were already weak prior to surgery. All five patients required a second treatment, which was provided within 24 hours of the first one. A third treatment was provided only to one patient with prolonged hospitalization due to surgery complications. This patient received a third treatment mainly for general pain, although mild shoulder pain was addressed too.

## 5. Discussion

In the present study, individualized acupuncture was found to be safe and a potentially efficacious adjuvant treatment to relieve pain in patients with PLSP. The employment of an individualized classical traditional Chinese medicine approach is unique in conventional care settings in which a disease oriented approach employing fixed formulas of acupuncture points is often utilized. The task of applying TCM syndromal diagnosis to patients with PLSP was indeed challenging in light of the relatively modern nature of this diagnosis. The acute changes induced by surgery and medications were not observed by TCM scholars at the time of canonization of TCM theory and practice. Consequently, the presentations of the syndromes were atypical and complex. In our study, we identified five major TCM syndromes (see Table 4) based on patient history and classical TCM physical examination. The rationalization of this process may serve researchers of other traditional therapies to construct treatment and research strategies in conventional care settings.

Although causality cannot be inferred in our observational study, we assume that the immediate outcomes, within 2 hours following treatment, were probably related to the acupuncture treatment. This assumption is derived from the natural history of PLSP, which usually takes a longer time to resolve [1, 2]. Moreover, patients were referred by physicians and the nursing staff to acupuncture treatment only if standard analgesics did not reduce pain sufficiently. This may reflect the severity and persistency of such pain, as well as the judgment of the medical staff regarding acupuncture's effectiveness for this indication. The effect attributed to acupuncture may be exerted by both the specific and nonspecific effects of an acupuncture session, and in this study we did not aim to differentiate between these components [26].

A possible limitation of this study is that the questionnaires were filled out by patients in their practitioners' presence. This may have inadvertently caused patients to “please” the practitioners by reporting better outcomes. Our study also lacks longer periods of follow-up. Repeated pain recordings on the days following treatment could provide such information.

Additional potential limitations are a carry-on or add-on effect of analgesics to that of acupuncture. It is truly difficult to differentiate between these effects; however, we do not find it necessary to do so. The concept of integrative medicine emphasizes making appropriate use of both conventional and complementary medicine; therefore, combining both for a patient with inadequate pain control fulfills this concept [27]. We are less interested in whether complementary medicine can, or should, replace other forms of conventional pain management but rather whether it can be used safely and effectively to complement current conventional pain management approaches. Differentiating between acupuncture and other cointerventions effect can be assessed using pragmatic trial methodology, that is, comparing between patients on analgesic use and acupuncture only. In addition, various treatment protocols can be established for various syndromes, and treatment outcomes per treatment protocols can be compared. The issue of appropriateness of individualized versus protocolized approaches in busy, high volume departments deserves attention. Based on our experience in such settings, we favor the former approach as long as detailed rationalization of treatment selection and documentation is provided. However, only future controlled trials comparing patient outcomes as well as economic analysis could possibly resolve this debate from both patient and administrative perspectives.

The novelty of our study is in the application of classical TCM approaches to modern acute hospital care. The TCM presentation of our patients was complex due to the physiological and biochemical effects of surgery and medications. Often, it was difficult to differentiate the underlying (presurgery) disharmony in TCM pattern diagnosis from the acute manifestations that were a consequence of the surgery. We therefore often selected to address both [25].

In summary, providing individualized acupuncture according to TCM principles seems to have potential in relieving PLSP. Randomized controlled trials should be conducted in order to validate our preliminary findings and differentiate between the effects of acupuncture and other cointerventions.

## Figures and Tables

**Table 1:** Patient characteristics.

Patients	Age (y)	Gender	Type of laparoscopic surgery
1	39	F	Hernia repair
2	40	F	Sleeve gastrectomy
3	50	F	Cholecystectomy
4	57	F	Sleeve gastrectomy
5	20	M	Sleeve gastrectomy
6	25	F	Cholecystectomy
7	53	F	Left hemicolectomy
8	58	F	Appendectomy
9	63	F	Hernia repair
10	37	F	Left hemicolectomy
11	22	F	Peritoneal lavage
12	52	F	Right hemicolectomy
13	71	M	Fundoplication
14	45	F	Cholecystectomy
15	57	F	Cholecystectomy
16	25	F	Sleeve gastrectomy
17	18	F	Sleeve gastrectomy
18	23	F	Sleeve gastrectomy
19	39	F	Cholecystectomy
20	45	F	Sleeve gastrectomy
21	20	M	Sleeve gastrectomy
22	61	M	Appendectomy + cholecystectomy
23	41	F	Gall bladder inflammation
24	27	F	Sleeve gastrectomy
25	22	F	Sleeve gastrectomy

**Table 2:** Levels of PLSP before and after treatment.

	Number of patients	Mean	Standard deviation	Minimum	Maximum	50th median
PLSP before	25	8.37	1.82	3.6	9.9	8.90
PLSP after	25	1.89	1.80	0.2	6.6	1.10

Note: *P* < 0.0001.

**Table 3:** Levels of general pain before and after treatment.

	Number of patients	Mean	Standard deviation	Minimum	Maximum	50th median
General pain before	24	6.44	2.53	0.0	8.9	7.40
General pain after	24	1.95	1.88	0.0	5.40	1.15

*P* < 0.0001.

One patient did not report general level of pain.

**Table 4:** Individual patient TCM syndrome and VAS scores.

Patient	Main TCM syndrome	PLSP before	PLSP after	General pain before	General pain after
1	Heat in the heart + phlegm	9.8	1.1	8.9	3.5
2	Sp Qi def. + phlegm	9.9	1.6	8.6	2.6
3	Phlegm	8	0.9	6.9	0.7
4	Phlegm	9.8	1.2	6.8	0.4
5	Lung Qi def.	9.9	3.8	7.8	2.1
6	Qi def. + phlegm	9.8	4.2	7.9	5.2
7	Phlegm heat in the stomach and intestine + kidney Yin def. + heat	7.9	0.9	5.7	2.8
8	Phlegm heat	3.6	0.6	5.4	0
9	Spleen Qi def., blood def. in the heart	9.9	1.9	1.2	0
10	Kidney Yin and Yang def.	5.2	1.1	0	0
11	Lung heat/fire + liver Yang rising	8.3	0.4	7.4	0.7
12	Spleen Qi def. + liver Qi stg.	9.7	0.3	6.6	1.2
13	Gall bladder heat and phlegm	7.1	1.8	0.8	0.3
14	Phlegm in upper burner	9.9	6.6	7.3	4.8
15	Liver Qi stg. + heat in upper burner	7	3.5	8.6	7.1
16	Lung heat/fire + gall bladder heat and phlegm stg.	8.9	1	6.6	0.4
17	Liver Qi and blood stg.	8	1	5.5	1.4
18	Kidney Yin and Yang def.	9.9	1.7	0	0
19	Stomach Qi stg. + spleen Qi def.	8.9	0.2	8.7	0.6
20	Spleen Qi def. + dampness + liver blood stg.	4.8	3.6	6	6.1
21	Spleen dampness + liver Qi stg.	9.7	6.5	6.8	4.8
22	Phlegm heat	9.7	0.8	8.8	1.2
23	Phlegm heat in the stomach + liver Qi stg.	6.3	0.6	4.7	0.8
24	Phlegm heat in the stomach + phlegm could in the triple burner	9.8	0.2	7.4	1.1
25	Spleen Qi def. and dampness, liver blood def.	7.6	1.8	5	1.6

**Table 5:** Acupuncture point selection according to TCM syndrome.

Syndrome	Key point
Phlegm	Pc5—a key point that was used every time that phlegm was diagnosed. St 40, Tw10, Liv 2, Gb 40—one or two points where combined depending on the TCM syndrome
Heat	Li 11, St 40, Liv 2, Gb 40, Sp 6, Kid 2.
Blood and Qi stagnation	Li4, Liv 3, Pc 6, Pc 7, Sp 10.
Dampness	Lu 5, Sp 9, Sp 6, Sp 3.
Blood and Qi deficiency	St 36, St 37, St 38, St 39, Sp 6, Liv 3, Liv 4, Sp, Sp 10.

The number of points chosen in each treatment ranged from four to eight.
